# Mental Health interventions on a group of Trafficked females, in Egypt

**DOI:** 10.1192/j.eurpsy.2025.1967

**Published:** 2025-08-26

**Authors:** F. S. A. M. Swilem, F. S. A. M. Swilem

**Affiliations:** 1 Child and Adolescent Psychiatry, Cambridge and Peterborough NHS Foundation Trust, Peterborough, United Kingdom

## Abstract

**Introduction:**

**Background:** The United Nations defines Human Trafficking as “the recruitment, transportation, transfer, harbouring, or receipt of people through force, fraud or deception, with the aim of exploiting them for profit,” and says it is practiced everywhere in the world. Studies show that women who have been trafficked for sex have higher levels of fear, are more isolated, greater trauma and mental health needs than other victims of crime, high rates of physical and sexual violence, memory loss, sexually transmitted diseases, and traumatic brain injuries. The present study attends to explore the mental health profile of a cohort of trafficked women from Egypt.

**Objectives:**

Studies show that women who have been trafficked for sex have higher levels of fear, are more isolated, greater trauma and mental health needs than other victims of crime, high rates of physical and sexual violence, memory loss, sexually transmitted diseases, and traumatic brain injuries.The present study attends to explore the mental health profile of a cohort of trafficked women from Egypt.

**Methods:**

This study included detailed mental health assessments of 42 trafficked women in and around Cairo in Egypt. Data was collected by social workers. Assessments, Diagnosis and interventions were done by the author with the help of two clinical psychologists.

**Results:**

This snapshot study shows very convincingly that there’s a high burden of mental ill health among the trafficked women.Suitable therapeutic interventions may provide effective management and prevention of further deterioration of mental health issues of these vulnerable trafficked women.

**Conclusions:**

This snapshot study shows very convincingly that there’s a high burden of mental ill health among the trafficked women.Suitable therapeutic interventions may provide effective management and prevention of further deterioration of mental health issues of these vulnerable trafficked women.

**Disclosure of Interest:**

None Declared

